# A nomogram to predict lymph node metastasis in patients with early gastric cancer

**DOI:** 10.18632/oncotarget.14660

**Published:** 2017-01-14

**Authors:** Chun Guang Guo, Dong Bing Zhao, Qian Liu, Zhi Xiang Zhou, Ping Zhao, Gui Qi Wang, Jian Qiang Cai

**Affiliations:** ^1^ Department of Abdominal Surgical Oncology, National Cancer Center/Cancer Hospital, Chinese Academy of Medical Sciences and Peking Union Medical College, Beijing, China; ^2^ Department of Endoscopy, National Cancer Center/Cancer Hospital, Chinese Academy of Medical Sciences and Peking Union Medical College, Beijing, China

**Keywords:** gastric cancer, lymph node metastasis, nomogram, endoscopic resection, decision analysis

## Abstract

**Background:**

Lymph node status is crucial to determining treatment for early gastric cancer (EGC). We aim to establish a nomogram to predict the possibility of lymph node metastasis (LNM) in EGC patients.

**Methods:**

Medical records of 952 EGC patients with curative resection, from 2002 to 2014, were retrospectively retrieved. Univariate and multivariate analysis were performed to examine risk factors associated with LNM. A nomogram for predicting LNM was established and internally validated.

**Results:**

Five variables significantly associated with LNM were included in our model, these are sex (Odd ratio [OR] = 1.961, 95% confidence index [CI], 1.334 to 2.883; P = 0.001), depth of tumor (OR = 2.875, 95% CI, 1.872 to 4.414; P = 0.000), tumor size (OR = 1.986, 95% CI, 1.265 to 3.118; P = 0.003), histology type (OR = 2.926, 95% CI, 1.854 to 4.617; P = 0.000) and lymphovascular invasion (OR = 4.967, 95% CI, 2.996 to 8.235; P = 0.000). The discrimination of the prediction model was 0.786.

**Conclusions:**

A nomogram for predicting lymph node metastasis in patients with early gastric cancer was successfully established, which was superior to the absolute endoscopic submucosal dissection (ESD) indication in terms of the clinical performance.

## INTRODUCTION

Early gastric cancer (EGC) has been increasing though overall incidence of gastric cancer declined around the world [1, 2]. According to Japanese Classification of Gastric Carcinoma (JCGC), EGC is defined as a lesion confined to the mucosa or the submucosa, regardless of the presence of lymph node metastases [3]. The outcome of EGC patients with D2 lymphadenectomy is excellent, with 5-year survival more than 90% [4]. As many as about 80% of patients exhibited no lymph node metastasis (LNM), most patients hence underwent excessive surgery and suffered from morbidity [5]. Efforts have been made to improve the quality of life for EGC patients, such as endoscopic submucosal dissection (ESD) [6], sentinel lymph node biopsy (SLNB) [7], or pylorus-preserving gastrectomy [8].

In East Asia, ESD has been accepted as an alternative to surgery and obtained a comparable long-term outcome [9]. Considering the risk of lymph nodal metastasis, only patients with differentiated mucosal adenocarcinoma, absence of lymphovascular invasion (LVI) and 20 mm or less in size are eligible for ESD (absolute indication) [3]. Given the excessively strict indication, various strategies were proposed to expand the ESD criteria for patients with negligible risk of LNM [10, 11]. Nevertheless, quantified prediction models for LNM based on individual information still remain absent, which is essential for clinicians to weigh treatment benefits and risks.

Nomogram is a graphic tool for individual probability of a clinical event based on a statistical predictive model. Increasing nomograms have been established for use in oncology. Two risk calculators developed from nomograms can efficiently identify the patients suitable for prostate cancer biopsy in a prospective multi-institutional study [12]. Through a nomogram for predicting bone metastasis in breast cancer, Delpech et al facilitated the selection of population at high risk for a virtual clinical trial [13]. However, nomograms for LNM in EGC patients have been rarely reported. In this study, we aimed to develop a nomogram for predicting lymph node metastasis for patient with EGC.

## RESULTS

### Demographics of EGC patients

Of the 952 patients, the average age was 55.3 ± 11.3 years, and 631 cases (66.3%) were male. Tumor size was 2.75 ± 1.66 cm. The number of lymph nodes harvested was 27.6 ± 11.0, and 175 patients (18.4%) were found lymph node involvement. Tumors of differentiated type, signet ring cell carcinoma (SRC) and other undifferentiated type were 37.1% (353), 17.8% (169) and 45.2% (430), respectively. Mucosal tumor was detected in 440 patients (46.2%), and LVI in 92 (9.7%).

### Univariate and multivariate analysis on LNM risk factors

In univariate analysis, sex (P < 0.001), tumor location (P = 0.003), depth of invasion (P < 0.001), tumor size (P < 0.001), histology type (P < 0.001) and LVI (P < 0.001) are closely related to LNM. Logistic regression modeling identified five variables to be significantly associated with LNM, including female sex (Odd ratio [OR] = 1.961, 95% confidence index [CI], 1.334 to 2.883; P = 0.001), submucosa (OR = 2.875, 95% CI, 1.872 to 4.414; P = 0.000), tumor size > 3cm (OR = 1.986, 95% CI, 1.265 to 3.118; P = 0.003), undifferentiated carcinoma types (OR = 2.926, 95% CI, 1.854 to 4.617; P = 0.000) and presence of LVI (OR = 4.967, 95% CI, 2.996 to 8.235; P = 0.000) (Table 1).

**Table 1:** Clinicolpathological factors associated with lymph node metastasis in patients with early gastric cancer

Variables	LNM			Multivariate	P
	Negative, n (%)	Positive, n (%)	P	Odds ratio (95% CI)	
Age, years					
≤60	517 (66.5)	113 (64.6)	0.619		
>60	260 (33.5)	62 (35.4)			
Sex					
Male	536 (69.0)	95 (54.3)	0.000*	1	
Female	241 (31.0)	80 (45.7)		1.961 (1.334 - 2.883)	0.001*
Location					
Upper third	140 (18.0)	13 (7.4)	0.003*	1	
Middle third	81 (10.4)	21 (12.0)		2.317 (1.013 - 5.300)	0.047*
Lower third	543 (69.9)	137 (78.3)		1.794 (0.931 - 3.458)	0.081
Whole	13 (1.7)	4 (2.3)		2.607 (0.629- 10.804)	0.186
Histology					
DC	320 (41.2)	33 (18.9)	0.000*	1	
SRC	148 (19.0)	21 (12.0)		1.239 (0.648 - 2.367)	0.517
Other UDCs	309 (39.8)	121 (69.1)		2.926 (1.854 - 4.617)	0.000*
Tumor size					
≤2cm	365 (47.0)	51 (29.1)	0.000*	1	
2~3 cm	221 (28.4)	57 (32.6)		1.599 (1.016 - 2.516)	0.043*
>3cm	191 (24.6)	67 (38.3)		1.986 (1.265 - 3.118)	0.003*
Macroscopic					
Elevated	142 (18.3)	33 (18.9)	0.938		
Flat	160 (20.6)	34 (19.4)			
Depressed	475 (61.1)	108 (61.7)			
Tumor depth					
T1a	402 (51.7)	38 (21.7)	0.000*	1	
T1b	375 (48.3)	137 (78.3)		2.875 (1.872 - 4.414)	0.000*
LVI					
No	739 (95.1)	121 (69.1)	0.000*	1	
Yes	38 (4.9)	54 (30.9)		4.967 (2.996 - 8.235)	0.000*
Ulceration					
No	731 (94.1)	165 (94.3)	0.917		
Yes	46 (5.9)	10 (5.7)			

LNM: lymph node metastasis; DC: Differentiated carcinoma; SRC: Signet cell ring carcinoma; UDC: Undifferentiated carcinoma; LVI: Lymphovascular invasion; CI: Confidence Interval. * P < 0.05.

### Nomogram for predicting lymph node metastasis in EGC patients

**Figure 1:**
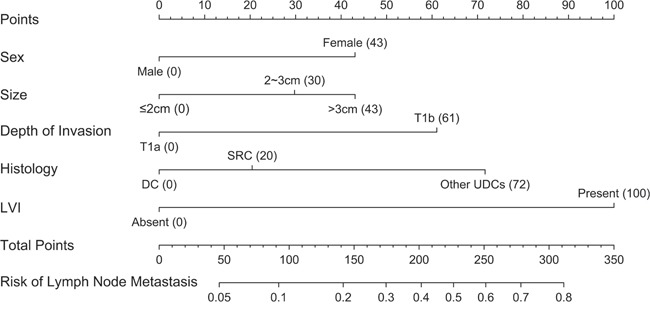
Nomogram for predicting lymph node metastasis in patients with early gastric cancer DC: Differentiated carcinoma; SRC: Signet cell ring carcinoma; UDC: Undifferentiated carcinoma; LVI: Lymphovascular invasion.

**Figure 2:**
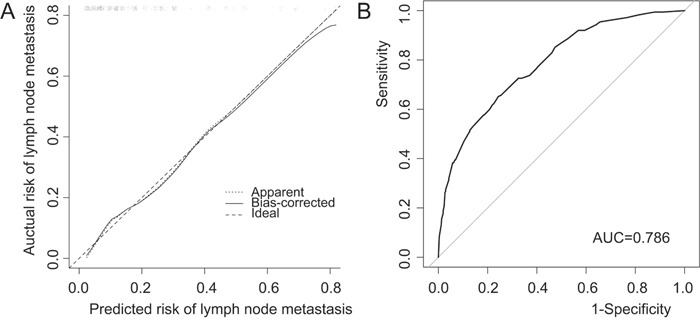
Validation of nomogram for predicting lymph node metastasis in early gastric cancer patients **A**. Calibration plot. After 500 repetitions, the bootstrap-corrected calibration curve (solid line) lay close to the ideal reference line (dashed line), which demonstrated a perfect agreement between the predicted and actual outcomes (mean absolute error = 0.013). **B**. Discrimination plot. After 2000 bootstrap repetitions, the receiver operating characteristic analysis demonstrated that area under curve was 0.786 (95% CI, 0.749 to 0.822).

A nomogram that incorporated the significant factors associated with LNM was constructed based on the logistic regression model (Figure 1). The nomogram confirmed LVI as the largest contributor to scores, followed by the histologic type and depth of tumor invasion. Tumor size and sex showed a modest impact on the model. Each level within variables was assigned a score according to the point scale. By summing up the total score and locating it on the total point scale, a corresponding probability of LNM for each individual was determined. The calibration plots presented a good agreement between the bias-corrected prediction and the ideal reference line with additional 500 bootstraps in Figure 2A (Mean absolute error = 0.013). The Hosmer-Lemeshow test resulted in a p value of 0.645, indicating that the model was well fitted. Figure 2B showed that the area under curve (AUC) for the nomogram to predict LNM was 0.786 (95% CI, 0.749 to 0.822). The estimated AUC of absolute indication for ESD was 0.554 (95% CI, 0.540 to 0.567).

### Clinical performance of absolute indication for ESD and the nomogram

**Figure 3:**
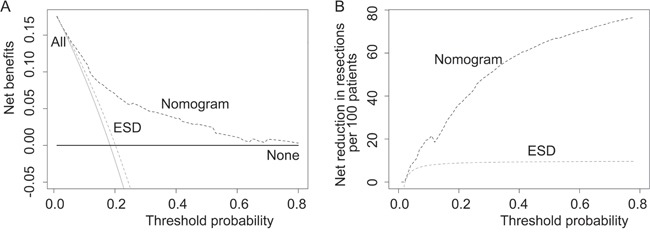
Clinical performance of absolute indication for ESD and the nomogram **A**. Decision curve analysis on absolute indication for ESD and the nomogram. The y-axis represents net benefits, calculated by subtracting the relative harms (false positives) from the benefits (true positives). The x-axis measures the threshold probability. A treatment strategy is superior if it has the highest value compared with other models, including two simple strategies, such as performing surgery for all patients (sloping solid line) or no patients (horizontal solid line). For example, the value of net benefits would be 0.115 if we select 10% as cutoff value, which means that nomogram would find about 11 patients with lymph node metastasis among one hundred patients compared with simple observation, without adding any unnecessary resections (false positives) theoretically. **B**. When assessing the clinical utility of two models, we sometimes are more interested in whether nomogram would reduce unnecessary gastrectomy. At the same cutoff of 10%, nomogram would reduce the unnecessary resection rate by 20%, without missing any cancers.

As concerned as the EGC patients, consequence of missing cancer is more important compared with the over diagnosis. Therefore, we focus on the relative value between false negative and false positive (termed net benefit) by decision curve analysis [14]. We examined the theoretical relationships for different models at a range of threshold probabilities (Figure 3 and Table 2). Compared with the two simple strategies of performing resection for every patient or no patients, the performance of nomogram exhibited an excellent net benefit over all the range of threshold probabilities. On the contrast, strategy based on the absolute indication only obtained tiny benefits than resection on every patient at range from 0% to 20%, and was inferior to the strategy of no resection after threshold of 20%. Herein, nomogram identified more patients with LNM than absolute criteria over most range of threshold probabilities except at 0-5%, without theoretically adding any false positive (Figure 3A). Meanwhile more reductions in unnecessary resection were exhibited in nomogram compared to ESD criteria after 3% (Figure 3B).

**Table 2:** Clinical performances between absolute criteria for ESD and the nomogram

Threshold Probability (%)	Net benefits per 100 patients			Net reduction in resections per 100 patients		Nomogram	
	Treat all	Nomogram	ESD	Nomogram	ESD	FNR (%)	NPV (%)
5%	14.1	14.6	14.4	9.2	5.7	2.3	97.6
7%	12.2	13.4	12.8	15.4	6.9	5.7	96.5
10%	9.3	11.5	10.2	19.9	7.8	11.4	94.9
15%	4.0	8.5	5.5	25.4	8.5	27.4	91.5
20%	-2.0	7.0	0.2	36.1	8.8	34.9	90.6

ESD: Endoscopic submucosal dissection; FNR: False negative rate; NPV: Negative predictive value.

According to absolute indication, 94 patients of the cohort (9.9%) were eligible for endoscopic treatment. The false negative rate (FNR) and false positive rate was 1.1% (2/175) and 88.2% (685/777), respectively.

## DISCUSSION

In this large retrospective study, we established a nomogram for predicting the risk of LNM in EGC patients, which demonstrated a good agreement between prediction and actual probability shown by discrimination and calibration plot. Moreover, the clinical performance of the nomogram was superior to absolute indication for ESD, which would keep more patients with negligible risk from excessive surgical resections.

Five variables associated with LNM were used for establishment of nomogram, including sex, tumor size, depth of invasion, histology and LVI, which were reported previously [15, 16]. The quantified prediction model allowed both clinicians and patients to take more objective decisions in treatment option. For example, a hypothetical male patient with a 1.5 cm signet ring cell carcinoma cancer, confined to mucosa, without LVI, may safely choose follow-up because of a low risk of 3.3% calculated by the nomogram. Conversely, a female patient with 3 cm undifferentiated submucosal cancer, classified as risk of 41.5%, should undergo an aggressive surgery with extended lymph node dissection.

As current standard for endoscopic treatment, absolute ESD indication showed an ideal FNR of 1.1% in this cohort. Most diagnostic models are evaluated with measures of accuracy, instead of addressing clinical consequences such as under- or over-diagnosis [17–19]. For instance, absolute indication could not be regarded as a good diagnose procedure because of a low discrimination power of 0.554, almost equivalent to chance. Herein, we focus on the comparison of clinical performances between models. As missing cancer is more harmful than overtreatment, we strive to weigh benefits (true positive) and harms (false positive) by decision analysis. The findings demonstrate that the nomogram gains more net benefits compared with ESD indication (Figure 3).

Until now, there has been no definite cutoff for predicted probability. The optimal threshold varies among clinicians, which mainly depends on how much the patients or clinicians reject the risk. In procedure of SLNB for breast cancer, 5% was usually used as an accepted FNR [20]. Fujikawa et al proposed two-thirds of clinical T1 gastric cancers would be suitable for endoscopic treatment in case FNR was 5% [21]. Similarly, the present model would identify 289 patients (30.4%) eligible for ESD with a FNR of 5.7% (Table 2). Different to breast cancer, it is worthy to note that no salvage treatment such as chemotherapy exists for EGC. The therapy for breast cancer was determined by multiple factors, such as primary lesion and hormone receptor, not the status of axillary lymph node alone [20]. Herein, a careful discussion with patients is indicated. However, nomogram will make choice easier under some special conditions, such as for elderly patients or those combined with severe comorbidity.

Quantified risk stratification probably is a good tool for selection of patients for individualized treatment. The use of SLNB in early gastric cancer remains debatable. The reported sensitivity of SLNB procedure varied from 40% to 100% among studies [7]. A meta-analysis with 46 studies showed the estimated sensitivity were 87.8% for SLNB, which may not be clinically useful due to its unsatisfactory sensitivity and significant inter-study heterogeneity [22]. Given the limitations in tissue sampling and sensitivity of technique, the FNRs might hardly reach zero though advance in molecular biological diagnosis [23]. Herein, selection of appropriate patients probably was the key to improve the performance of SLNB procedure. In theory, we maybe accept a “not bad” clinical outcome for a population at low risk when a procedure with high FNR was applied. Supposing FNR of SLNB examination was 10%, we would observe maximum 3 cases falsely missed in a subgroup with a risk of 20-30%. Ninety-seven patients who should undergo additional gastrectomy will benefit from the strategy. In future, strategy based on nomogram maybe play a big role in selection of patients for individualized treatment.

Little studies were reported on nomogram predicting lymph node metastasis in EGC patients. Recently, Zheng et al [17] firstly developed a model to predict LNM based on eight variables, with a better discrimination ability of 0.860 than ours. However, the discrimination power of nomogram should be interpreted cautiously when evaluated in different datasets. For example, the Memorial Sloan Kettering nomogram, a model for predicting non-sentinel lymph node involvement in breast cancer, reported an AUC of 0.77 [24], which yielded various AUCs ranging from 0.56 to 0.72 in different series [25–27]. The true value of the nomogram for a particular patient will be truly manifested only when applied to a cohort with similar patients and disease characteristics [28].

Several limitations exist in the present study. As advance in endoscopic technique and instrument, the expanded ESD has been widely performed in most high-volume centers. However, incomplete measure data of invasion depth hinders the further evaluation in this retrospective study. To evaluate the value of the nomogram in the eligible patients with expanded indication, we performed a virtual comparison according to the depth of invasion (see Supplementary Figures 1 and 2). Consequently, the nomogram seemed to demonstrate a more favorable performance to expanded indication. Of course, the results still need to be confirmed in a real dataset. Next, this nomogram was only internally validated in single center using bootstrapped calibration, which might be biased by institutional diagnostic patterns. The further evaluation in external datasets or prospective study was indicated. At last, the discrepancy between ESD and the surgical pathology in the histology reports was deserved to be noticed. The incidence of LVI was more prevalent in the specimen of ESD than surgery because of the thinner section (2 mm vs. 5 mm).[3] And LVI detected by immunohistochemical staining (IHCS) was about ten times as those by hematoxylin-eosin staining (HES) despite the clinical significance was still unclear.[29] Herein, there was an underestimate of the incidence of LVI in the surgical specimens, which perhaps could explain why LNM occurred in the patients with no risk factors. As discussed above, this nomogram is mainly applied to the patients after ESD to decide whether additional surgery is required or not. The patients with LVI after ESD have more chance to receive an aggressive gastrectomy because of a high score calculated by the nomogram. Consequently, this nomogram derived from surgical specimens still needs further validation in those after ESD.

In conclusion, we established a nomogram for predicting lymph node metastasis in patients with early gastric cancer. The nomogram was superior to the absolute ESD indication in terms of the clinical performance.

## MATERIALS AND METHODS

The study was approved by Institutional Ethical Board of Cancer Hospital, Chinese Academy of Medical Sciences. From January 2002 to December 2014, 1,494 EGC patients underwent curative resection with D2 lymphadenectomy in our hospital. Patients were excluded if they had neoadjuvant chemotherapy, resident gastric cancer, no lymphadenectomy, incomplete medical information, and other coexisting tumors or less than 15 lymph nodes examined. A total of 952 patients were enrolled in the study.

Gastrectomy was performed as described [30], which involved resection of at least two-thirds of the stomach with a D1 or D2 lymph node dissection. A gross resection margin of more than 2 cm was ensured. For tumors adjacent to the esophagus or duodenum, frozen section biopsy of the margin was examined to ensure a R0 resection. Preoperative endoscopic marking by clips or blue dying was indicated if tumor was considered as cT1. Curative resection was defined as the absence of cancer in both the upper and lower resection margins and no evidence of residue lesions. Surgical specimens were assessed by two advanced pathologists as recommended as Japanese classification of gastric carcinoma [3]. Series sections along the lesser curvature were made at 3 to 4 mm intervals and each section was sliced into 4 μm in thickness. Lymph nodes dissected from the specimen were fixed in a 10% buffered formalin solution. Each harvested lymph node was examined by spiting in half along the maximum diameter and stained with H&E section. Immunohistochemical staining, such as HER2 and TOP2A, was not performed until 2009. No immunohistochemical staining for LVI were used.

The clinicopathological variables, including sex, age, tumor size, depth of invasion, macroscopic type, histology, lymphovascular invasion, and ulceration, were obtained from a prospective database. Tumor histology was classified as recommended as JCGC: differentiated carcinoma (DC), which included papillary adenocarcinoma, and well or moderately differentiated adenocarcinoma; and undifferentiated carcinoma (UDC), including poorly or undifferentiated adenocarcinoma, SRC and mucinous carcinoma [3]. The macroscopic appearance was analyzed in such types: elevated type (I and IIa), flat type (IIb), or depressed type (IIc and III). Lymph node metastasis and depth of tumor invasion were defined according to the American Joint Committee on Cancer (AJCC) staging [31]. Lymphovascular invasion was defined as presence of tumor emboli either in lymphatic duct or vascular lumen [3].

### Statistical analysis

Descriptive data are presented as mean ± SD. For comparisons between different groups, continuous variables are analyzed using the Student's t test, and categorical variables were analyzed using chi-square test. Factors significant in univariate analysis are included in multivariate logistic regression analysis to identify independent variables. The performance of the established logistic regression model was internally validated with bootstraping analysis. We evaluated the discrimination power of the nomogram by calculating the concordance index, which is identical to the nonparametric area under the receiver operating characteristic curve. AUC ranges from 0 to 1, with 1 indicating perfect concordance, 0.5 indicating no better concordance than chance. To test the significance of the AUC, we created 2000 concordance indices for the model by using bootstrapping analysis and obtained 95% confidence interval (CI). Subsequently we constructed a plot of calibration, with additional 500 bootstrap samples to reduce the overfit bias. Finally, a decision curve analysis described by Vickers et al was performed to assess the clinical utility of models by quantifying the net benefits when different threshold probabilities were considered [14]. The Statistical Package for the Social Sciences (SPSS) for Windows, Version 18.0 (SPSS Inc., Chicago, IL, United States) or the rms package and pROC package in R version 3.2.2 were used in this study [32]. All tests were two-sided and p value less than 0.05 was considered statistically significant.

## SUPPLEMENTARY MATERIALS FIGURES AND TABLES


