# Supplementary material for: A nomogram to predict lymph node metastasis in patients with early gastric cancer
Source: Oncotarget. 2017 Jan 14;8(7):12203–10. doi: 10.18632/oncotarget.14660 (PMC5355337; doi:10.18632/oncotarget.14660)
Supplement: Supplementary file 1 [file oncotarget-08-12203-s001.pdf]

A nomogram to predict lymph node metastasis in patients with early gastric cancer

SUPPLEMENTARY DATA

According to Japanese gastric cancer treatment guidelines, the resection is considered as curative when all of the following conditions are fulfilled (expanded indication): En-bloc resection, both horizontal and vertical margin negative, LVI(-), and (a) tumor size >2 cm, differentiated, pT1a, negative ulcerative findings (UL), or (b) tumor size no more than 3 cm, differentiated, pT1a, UL (+), or (c) tumor size no more than 2 cm, undifferentiated, pT1a, UL (-), or (d) tumor size no more than 3 cm, differentiated, pT1b (SM1, <500 μm from the muscularis mucosae). To simulate the comparison between the nomogram and the expanded indication of ESD, we established two groups, including ExpandedESD1 group and ExpandedESD2 group. ExpandedESD1 group was composed of the patients who meet with the expanded indication of ESD **except** those with tumor size no more than 3 cm, differentiated, no submucosal invasion deeper than 500 μm from the muscularis mucosa. 264 patients were classified as low risk in ExpandedESD1 group (264/952) and false negative rate (FNR) was 6.9% (12/175). ExpandedESD2 group was composed of ExpandedESD1 group and those with tumor size no more than 3 cm, differentiated and **pT1b**, which included 387 patients (387/952) with low risk and FNR was 14.3% (25/175). We speculated that FNR of the expanded indication of ESD varied between ExpandedESD1 and ExpandedESD2. Supplementary Figure S1 revealed the nomogram (black line) gained the most net benefits at most threshold. Moreover, this model avoided the unnecessary gastrectomy as much as possible in theory (Supplementary Figure S2).

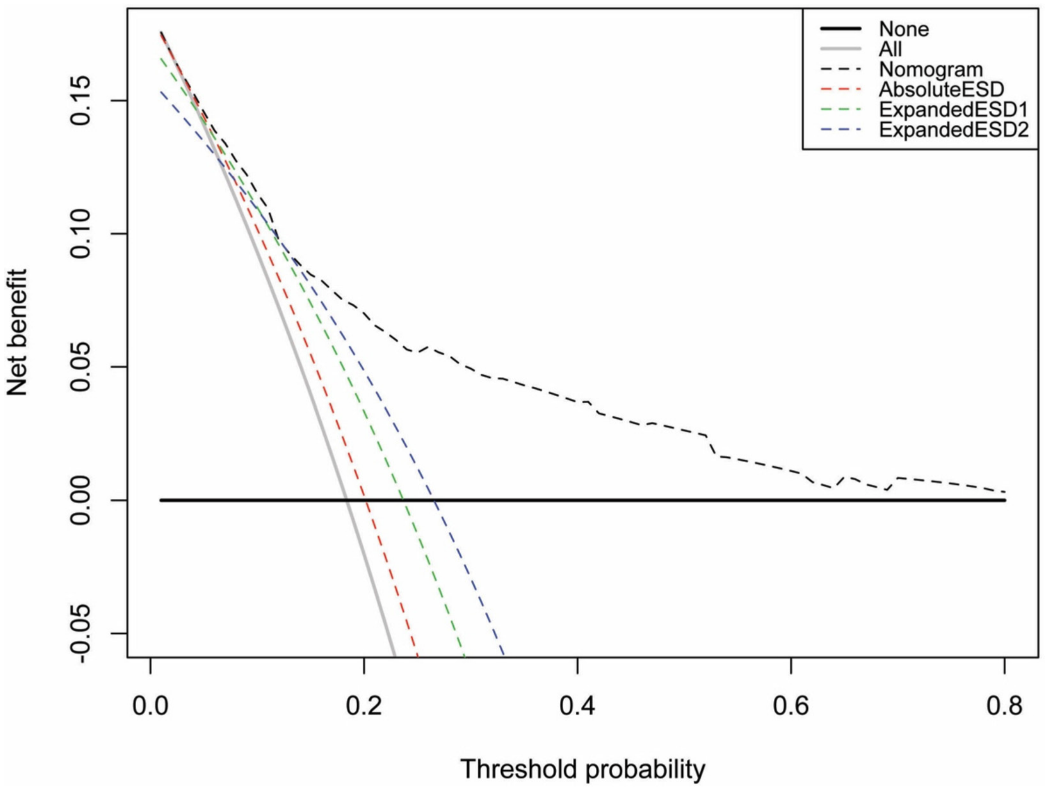

Supplementary Figure 1: Clinical performance of multiple risk models for LNM of EGC. Expanded ESD1: the patients meet with the expanded indication for ESD except those with tumor size no more than 3 cm, differentiated, pT1b (SM1, <500 μm from the muscularis mucosae). ExpandedESD2: the patients including the ExpandedESD1 and those with tumor size no more than 3 cm, differentiated and pT1b.

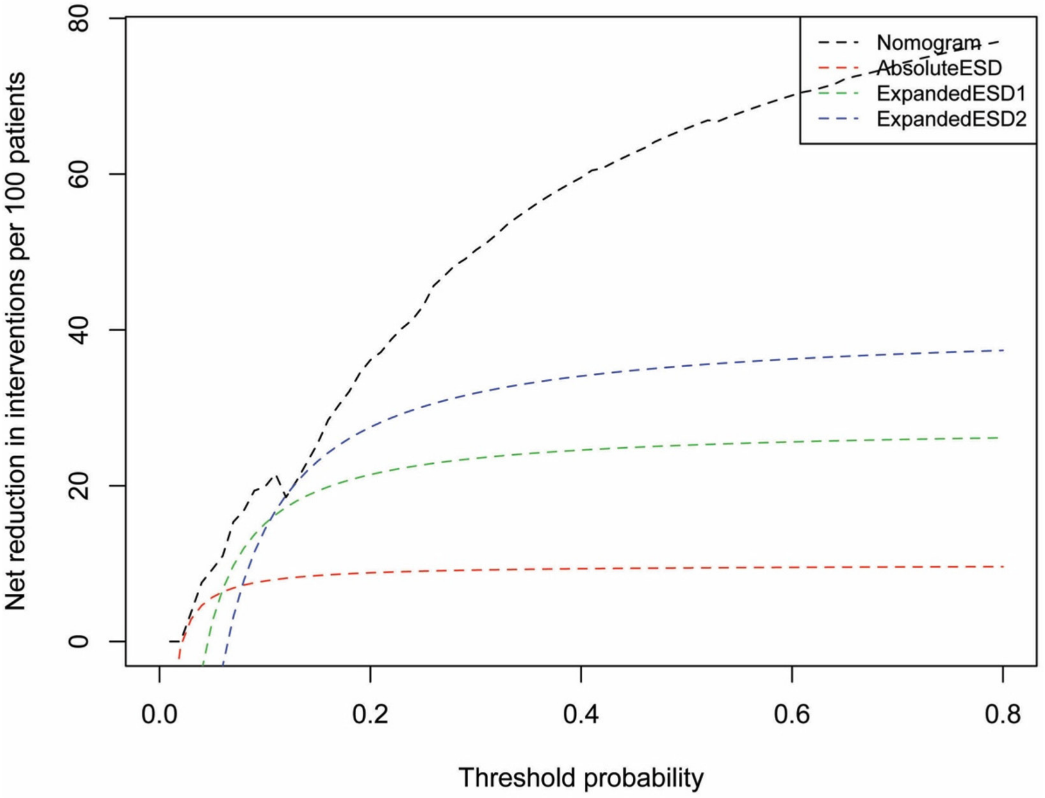

Supplementary Figure 2: Clinical performance of multiple risk models for LNM of EGC. Expanded ESD1: the patients meet with the expanded indication for ESD except those with tumor size no more than 3 cm, differentiated, pT1b (SM1, <500 μm from the muscularis mucosae). ExpandedESD2: the patients including the ExpandedESD1 and those with tumor size no more than 3 cm, differentiated and pT1b.
